# MEFV Gene Profile in Northwest of Iran, Twelve Common MEFV Gene Mutations Analysis in 216 Patients with Familial Mediterranean Fever

**Published:** 2015-01

**Authors:** Farhad Salehzadeh, Mehdi Jafari Asl, Saeid Hosseini Asl, Sepideh Jahangiri, Shahram Habibzadeh

**Affiliations:** 1 Departments of Pediatrics, Bouali Hospital, Ardabil University of Medical Sciences, Ardabil, Iran;; 2 Molecular-Genetic Laboratory, Imam Khomeini Hospital, Ardabil University of Medical Sciences, Ardabil, Iran;; 3 Department of Infectious Diseases, Bouali Hospital, Ardabil University of Medical Sciences, Ardabil, Iran

**Keywords:** Familial Mediterranean Fever, MEFV gene, Iran, Tel-Hashomer criteria

## Abstract

Familial Mediterranean Fever (FMF) is a hereditary autoinflammatory disease with autosomal recessive inheritance pattern often seen around the Mediterranean Sea. It is characterized by recurrent episodes of fever and polyserositis and rash. Recently, MEFV gene analysis determines the definitive diagnosis of FMF. In this study, we analyzed 12 MEFV gene mutations in more than 200 FMF patients, previously diagnosed by Tel-Hashomer clinical criteria, in northwest of Iran, located in the proximity of the Mediterranean Sea. In the northwest of Iran (Ardabil), 216 patients with FMF diagnosis, based on Tel-Hashomer criteria, referred to the genetic laboratory to be tested for the following mutations; P369S, F479L, M680I(G/C), M680I(G/A), I692del, M694V, M694I, K695R, V726A, A744S, R761H, E148Q. All patients were screened for MEFV gene mutations by a reverse hybridization assay (FMF Strip Assay, Vienna lab, Vienna, Austria) according to manufacturer’s instructions. Among these FMF patients, no mutation was detected in 51 (23/62%) patients, but 165 (76/38%) patients had one or two mutations, 33 patients (15/28%) homozygous, 86 patients (39/81%) compound heterozygous and 46 patients (21/29%) were heterozygous. The most common mutations were M694V (23/61%), V726A (11/11%) and E148Q (9/95%) respectively.

MEFV gene mutations showed similarities and dissimilarities in different ethnic groups, while it is common among Arabs and Armenians genotype. Since common 12 MEFV gene analysis could not detect up to 50% of our patients, who had FMF on the basis of clinical Tel-Hashomer criteria, clinical criteria is still the best way in the diagnosis of FMF in this area.

*The abstract of this article has been presented in the 7th Congress of International Society of Systemic Auto-Inflammatory Diseases in Lausanne, Switzerland, 22-26 May 2013.*

## Introduction

Familial Mediterranean fever (FMF) is a hereditary recurrent inflammatory disease with autosomal recessive pattern of inheritance characterized by self-limiting recurrent fever and pain in the serous membranes, pleurisy, peritonitis, arthritis and erythema.^1^^,^^2^ The most important complication of FMF is amyloidosis that eventually leads to kidney failure.^3^ Symptoms can occur in the first 10 years of life and according to the recent study in more than 80 percent of patients, the symptoms appear in childhood era.^1^

FMF is often presented in people of Mediterranean ancestry.^4^ The disease is mostly seen among Turks, Arabs, Armenians, and Jews.^5^Gene associated with the disease (Mediterranean Fever) MEFV gene was detected in 1997, it is located on the short arm of chromosome 16(16p13). The MEFV gene encodes pyrin, a protein expressed in neutrophils, eosinophils, monocytes, dendritic cells, and ﬁbroblasts. In monocytes, pyrin co-localized with the microtubule system. This fact may contribute to the therapeutic effect of colchicine, which destabilizes the cytoskeleton. The exact role for pyrin and the mechanisms by which MEFV mutations (more than 300 variants) exert their pathogenic effects are inadequately understood. Initial diagnosis of FMF is mainly based on clinical Tel-Hashomer criteria, as described below.^6^

Definitive diagnosis requires at least two major criteria or one major and two minor criteria:

*Major criteria*

- Fever with peritonitis, synovitis, or pleurisy
- Recurrent febrile attacks
- AA amyloidosis (without risk factors or other chronic inflammatory diseases)

Minor criteria

- Erysipeloid erythema
- Response to colchicine
- Family history of periodic syndrome

While identifying MEFV gene mutations determines the definitive diagnosis of FMF, clinical manifestations of the disease is much more sensitive and specific to diagnosis, especially in practice. It should be kept in mind that failure to find mutations in the MEFV gene does not rule out the disease.^6^

Bonyadi, in a cohort of 200 unrelated healthy individuals, screened for the five most common MEFV mutations (M694V, V726A, M680I, M694I, and E148Q). In this genotyping, the carrier rate among the Azeri people was 25.5%, with E148Q (11.5%) followed by V726A (1.75%). This study showed that E148Q mutation and carrier rate are high among the Iranian-Azeri people. The remaining common mutations were not found in this cohort.^3^

The allele frequency of MEFV gene mutations has high heterogeneity among the Turkish population and they are believed to be a high-risk population for developing FMF. The carrier-state frequency of FMF is 20%. The ﬁve most common mutations in the MEFV gene are M694V, E148Q, M680I, V726A, and M694I.^7^

This study is conducted to evaluate 12 common MEFV gene mutations in FMF patients (with documented FMF) to design a common MEFV gene profile. Additionally, we aimed at evaluating the diagnostic value of Tel-Hashomer clinical criteria versus genetic study in patients living in the northwest of Iran (near the Mediterranean Sea) having Azeri-Turkish background. 

## Materials and Methods

Based on the Tel-Hashomer clinical criteria, 300 FMF diagnosed patients enrolled in this descriptive case series study during August 2011 to September 2012. The study took place at a FMF clinic in northwest of Iran and the participants were selected among the Azeri-Turkish community in Ardabil. In total, 216 patients participated in the study; among which 84 patients were unavailable, unwilling, or not prepared to fill-in the consent form. This study was approved by the Ethical Committee of the Medical Faculty.

The patients were investigated and screened for the 12 known FMF mutations by a reverse hybridization assay (FMF StripAssay, Vienna lab, Vienna, Austria) according to manufacturer’s instructions. About 100 ml of peripheral blood was used for extracting DNA by boiling-based method. Initially, exons 2, 3, 5 and 10 were amplified for each patient in a single and multiplex PCR, with the primers supplied in the RDB kit. The thermocycling program of amplification was 35 cycles (94ºC for 15 seconds, 58ºC for 45 seconds, and 72ºC for 45 seconds) and a final extension at 72ºC for 7 minutes. Agarose electrophoresis revealed the accuracy of the amplification by detecting four amplified DNA fragments, including 206, 236, 295, and 318 bp. In a test strip presenting a parallel array of allele-specific oligonucleotide probes, biotinylated PCR products were selectively hybridized. By this means, the twelve common mutations E148Q in exon 2, P369S in exon 3, F479L in exon 5 and M680I (G/C), M680I (G/A), I692del, M694V, M694I, K695R, V726A, A744S, and R761H in exon 10 were determined.

Statistical analysis was performed using SPSS V 15.0. Comparison between the different genotypes was assessed using a chi-square test. P=0.05 was accepted as statistically significant. 

## Results

Among the 216 patients, 121 patients (56.01%) were male and 95 patients (43.99%) were female. The mean age of patients was 19-59 years and with a range from young infant (3 months) to 74 years of age. In this study, abdominal pain in 205 (95%), fever in 200 (93%), arthritis in 9 (4.33%), and chest pain in 5 (2.33%) were observed. 51 patients (23.62%) had no mutation, 165 patients (76.38%) have one or two mutations. Among these, 33 patients (15.28%) were homozygous, 46 patients (21.29%) heterozygous and 86 patients (39.81%) were compound heterozygous. As shown in table 1, thirty different genotypes were identified. Five mutations, M694V (23.61%), V726A (11.11%), E148Q (9.95%), M680I (9.02%) and R761H (3.47%) were the most common mutations in this study (table 2). The I692del, V761R, R726A mutations were rare and only detected in three patients. There was no significant difference between results for male and female; however, there were some similarities and dissimilarities in comparison with other studies (table 3-5).

**Table 1:** Distribution of the MEFV gene mutations

**Mutation**	**Genotype**	**Number**	**%**
Heterozygote	E148Q	18	8.33
(n=46)	M694V	14	6.48
(21/29%)	V726A	4	1.85
	A744S	4	1.85
	M680I	3	1.38
	P369S	2	0.92
	R761H	1	0.46
Homozygote	M694V	18	8.33
(n=33)	M680I	9	4.16
(15/28%)	E148Q	3	1.3
	M694I	1	0.46
	V726A	1	0.46
	R761H	1	0.46
Compound heterozygote	M694V-V726A	24	11.11
(n=86)	E148Q-M694V	11	5.09
(39/81%)	M680I-V726A	8	3. 70
	R761H-M694V	8	3.70
	M680I-M694V	5	2.31
	V726A-R761H	5	2.3
	M694V-M694I	4	1.85
	M680I-R761H	4	1.85
	E148Q-P396S	3	1.38
	M694V-P396S	3	1.38
	K694R-V726A	2	0.92
	A744S-R761H	2	0.92
	E148Q-V726A	2	0.92
	V726A-M694I	2	0.92
	M680I-M694I	1	0.46
	M694I-R761H	1	0.46
	I162del-R761H	1	0.46
Patients with no identified mutation		51	23.62

**Table2:** The most common MEFV gene mutation frequency

**Mutation**	**Number of alleles**	**Frequency (%)**
M694V	102	23.61
V726A	48	11.11
E148Q	43	9.95
M680I	39	9.02
R761H	15	3.47
M694I	13	3

**Table 3:** Mutations in different studies

**Study**	**Number of** **Patients**	**Mutation frequencies (%)**			**Number of mutations**
		**Heterozygous**	**Compound Heterozygous**	**Homozygous**	
Dundar^7^	2067	26.41	15.29	8.60	49.60
Yilmaz^9^	78	28	36	32	4
Esmaeili^8^	190	22.10	19.47	21.57	36.84
Bonyadi^10^	524	21.37	28.81	17.17	32.65
Balci^11^	25	18	24	12	46
Our study	216	21.29	39.81	15.28	23.62

**Table 4:** Comparison of allele frequencies in similar studies

**Study**	**Mutation frequencies (%)**				
	**M694V**	**V726A**	**M680I**	**M694I**	**E148Q**
Dundar^7^	14.68	4.76	7.62	0.46	5.15
Yilmaz^9^	55	3	16	0	10
Balci^11^	38	4	8	0	4
Bonyadi^10^	42.4	17	15.2	2.2	16.2
Esmaeili^8^	28	9	7	1	7
Our study	23.61	11.11	9.02	3	9.95

**Table 5:** Frequency of the fivemost common MEFV gene mutations among different ethnic groups

**Ethnic group**	**M694V**	**V726A**	M680I*	**M694I**	**E148Q**	Overall frequency (%)**
Jews	77	12.3	0.6	0	10.2	51
Armenians	52	26	20	0.2	1.8	94
Arabs	42.5	23.1	9.6	14.1	10.7	47
Turks	71.3	8.5	15	1.7	3.5	68
Iranian-Azeri Turks	54	16.7	12.6	2.5	14.2	52
Our study	23.61	11.11	9.02	3	9.95	57

*In some references, both G to C and G to A were studied; **The fraction of identifiable mutations among all independent alleles regarding the five common mutations

## Discussion

In 216 FMF patients, 51 patients (23.62%) had no mutations, 46 patients (21.29%) heterozygous, 86 patients (39.81%) compound heterozygous and 33 patients (15.28%) were homozygous (table 2). The most common mutation was M694V (23.61%) and in the second place was the V726A (11.11%), as shown in table 2.

In comparison with different ethnic groups as well as different studies, the 12 common MEFV gene mutations show similarities and dissimilarities (table 3 and 4). In all ethnic groups, M694V was the most common mutation. M680I was the second most common mutation among the Turks, whereas V726A was the second mutation among the Jews, Armenians, Arabs, and Iranian-Azeri. While M6941 is common among Arabs and least frequent among Jews, it displayed a higher rate among the Iranian-Azeri. Except E148Q mutation, it seems that Armenians and the population in our study have the same pattern of mutations, although the frequency of MEFV gene is similar to Arabs.

Bonyadi showed high frequency of E148Q in normal Azeri people, but our study represents significant frequency of this mutation in FMF patients as well (table 5).^8^

In this study, common compound mutations were M694V-V726A (11.11%), M694V-M694V (8.33%), and E148Q-M694V (5.09%) respectively. However, in other studies^7^^-^^10^ M694V-M694V is the most common mutation. Turkish researchers^7^^,^^9^showed that M694V-M680I is the second common compound mutation, however, Azeri-Turkish studies^8^^,^^10^showed that M694V-V726A is the second common compound mutation.

In the present study, we rarely observed V761R, R726A, and I692del with one mutation. Furthermore, there are no reports on the compound heterozygote mutations K694A/V726A, M694I/R761H, I692del/R761H, and A744S/R761H, as the case in our patients. The key point in this study is the re-evaluation of MEFV gene in the diagnosis of FMF since we could only detect 55.09% of those patients suffering from FMF (by 12 common MEFV gene analysis). It seems that clinical criteria are still the best method in the diagnosis of FMF. In addition, molecular analysis of FMF mutations in Chetrit’s study confirmed the diagnosis in about 60% of the referrals with suspected FMF.^12^Some individuals with paired MEFV mutations do not have clinical symptoms^13^ and one molecular study showed diagnosis of FMF was unlikely in 7% of patients based on clinical criteria.^14^

Many diagnostic criteria for FMF were initially developed for adults (Tel-Hashomer criteria and Livneh criteria) and then for children (Yalcinkaya criteria). All criteria have high sensitivity, but low speciﬁcity; particularly in the pediatric age group where recurrent fever attacks are more common than in adults. Since the identiﬁcation of the MEFV gene, considerable progress has been made in the understanding of FMF. To date, more than 300 sequence variants (mutations and polymorphisms) have been identified in MEFV gene. Gene analysis test is a valuable diagnostic tool, but is still unable to conﬁrm diagnosis in all patients. Consequently, more attention should be given to the clinical diagnostic criteria.^15^As a whole, a new moleculo-clinical approach to FMF is inevitable in the future.

## Conclusion

M694V is the most common mutation, and M694V-V726A is the most common compound in northwest of Iran. MEFV gene frequency is similar to studies on Arabs and Armenians. It is concluded that clinical criteria are the best way in FMF diagnosis. 
